# “Our interventions are still here to support communities during the pandemic”: Resuming mass drug administration for neglected tropical diseases after COVID-19 implementation delays

**DOI:** 10.1371/journal.pntd.0011368

**Published:** 2023-06-26

**Authors:** Tikhala Itaye, Sultani Hadley Matendechero, Jean Bosco Mbonigaba, Fikre Seife Gebretsadik, Tuduetso L. Molefi, Gilbert Baayenda, Eugene Ruberanziza, Karsor K. Kollie, January Zilabumba, Massitan Dembele, Kebede Deribe, Elia Muhima Adrien, Maria Rebollo Polo, On behalf of the Kikundi African NTD Program Managers Community of Practice

**Affiliations:** 1 Department of Global Health, University of Washington, Washington, Seattle, United States of America; 2 National Public Health Institute, Ministry of Health, Nairobi, Kenya; 3 Rwanda NTD and Other Parasitic Diseases Program, Rwanda Biomedical Centre, Ministry of Health, Kigali, Rwanda; 4 NTD Programme, Ministry of Health, Addis Ababa, Ethiopia; 5 NTD Programme, Ministry of Health, Gaborone, Botswana; 6 NTD Programme, Ministry of Health, Kampala, Uganda; 7 The END Fund, New York, New York City, United States of America; 8 NTD Programme, Ministry of Health, Monrovia, Liberia; 9 Technical Advisor, Ministry of Health, Zanzibar, Tanzania; 10 National Lymphatic Filariasis Elimination Program, Ministry of Health, Bamako, Mali; 11 Expanded Special Project on the Elimination of Neglected Tropical Diseases, World Health Organization -African Region, Brazzaville, Republic of Congo; University of Buea, CAMEROON

## Abstract

The COVID-19 pandemic disrupted essential health services, including those provided by national neglected tropical disease (NTD) programs. Most mass drug administration (MDA) programs were postponed for 6–12 months following World Health Organization guidance released in April 2020 to temporarily halt NTD programs and launch necessary COVID-19 precautions. While NTD-endemic countries have since resumed MDA activities, it is critical to understand implementers’ perspectives on the key challenges and opportunities for program relaunch, as these insights are critical for maximizing gains towards disease control and elimination during public health emergencies. Using data from using online surveys and focus group discussions, this mixed-methods study sought perspectives from Ministry of Health NTD Program Managers and implementing partners from non-governmental organizations working in sub-Saharan Africa. Data analysis revealed that findings converged around several main themes: disruptions for MDA programs included resource shortages due to prioritization of pandemic response, challenges adhering to COVID-19 safety protocols, and community hesitancy due to coronavirus transmission fears. Identified solutions for restarting MDA programs focused on adapting intervention delivery and packaging to minimize disease transmission, embracing technology to optimize intervention planning and delivery, and identifying opportunities to promote program integration between pandemic response strategies and NTD campaign delivery. Findings identifies key challenges due to disruptions to NTD program delivery and provide strategic recommendations for endemic countries to build resilient programs that can continue to perform during and beyond global pandemics.

## Author summary

In April 2020, global guidelines suspended mass drug administration (MDA) programs used in the control and elimination of neglected tropical diseases (NTDs) due to the ongoing COVID-19 pandemic. While the guidance aimed to reduce the risk of coronavirus exposure and transmission among stakeholders involved in MDA delivery and recipient community members, it risked impeding the gains made by MDA programs towards NTD control and elimination, leaving billions at risk of these infectious diseases. This study summarizes the perspectives of Ministry of Health NTD Program Managers and representatives from non-governmental organizations across sub-Saharan African working in NTDs regarding challenges imposed on MDA programs by the COVID-19 pandemic and identify opportunities to improve planning and implementation during the relaunch of MDA programs. Respondents noted key disruptions for MDA programs, including resource shortages, challenges adhering to COVID-19 safety protocols, and community hesitancy due to coronavirus transmission fears. Specific solutions for restarting MDA programs included adaptations to MDA strategies to incorporate safer, low-contact drug delivery techniques, incorporating technology to optimize MDA planning and delivery, and identifying opportunities to promote integration between NTD campaign delivery and pandemic response strategies. This paper includes suggestions for building resilient programs that can continue to perform in the face of public health emergencies.

## Introduction

Neglected tropical diseases (NTDs) are a group of highly debilitating diseases affecting more than one billion people globally–approximately one-sixth of the world’s population [1]. These diseases prevail in tropical and sub-tropical regions and thrive in areas with limited access to adequate sanitation, clean water and healthcare, with Africa carrying almost 40% of the global NTD burden [2,3]. Five NTDs–lymphatic filariasis, onchocerciasis, soil-transmitted helminths, schistosomiasis, and trachoma–are controlled through mass drug administration (MDA) of preventative chemotherapy (PC), which entails distributing safe and effective drugs to all individuals living in NTD-endemic areas, regardless of infection status. MDA campaigns for these PC-NTDs typically include fixed-point delivery mechanisms, such as school-based distribution by teachers or door-to-door delivery by volunteer community drug distributors (CDDs) [4].

In April 2020, the World Health Organization (WHO) suggested temporarily halting MDA programs to reduce the risk of transmission of COVID-19 amongst stakeholders involved in MDA delivery and recipient community members [5]. The guidelines recommended temporary cessation of key MDA-related activities including case detection, community surveys, and mass treatment. At the time of the pause in MDA activities, countries in sub-Saharan Africa had recorded more than 200,000 COVID-19 cases and many countries had already instituted precautionary measures such as lockdowns and physical distancing, further restricting intervention delivery activities for regions that had planned to conduct MDA [6].

During the pandemic, WHO released a new NTD road map providing ambitious goals for the control and elimination of the five PC-NTDs [7]. These new guidelines were released only six months after WHO published risk assessment tools to guide Ministry of Health (MOH) NTD Program Managers (PMs) and implementation partners in restarting planned MDA program activities and precautionary safety measures to mitigate the risk of COVID-19 transmission [5]. While exhaustive in detailing the necessary decision-making frameworks necessary for MDA program continuation, the risk assessment tool did not include specific recommendations regarding pandemic-specific modifications to MDA planning and implementation activities. Given the considerable concern for a potential recrudescence of transmission due to COVID-19 related disruptions in MDA implementation and evaluations, it is critical that any recommendations for restarting programs align with implementer perspectives. Thus, this study aimed to understand how COVID-19 influenced MDA programming and identify innovative ideas for improving the resumption of implementation activities from the perspective of key NTD stakeholders. Findings aim to provide implementer perspective on opportunities to overcome challenges related to the COVID-19 pandemic during the relaunch of MDA programs.

## Methods

### Ethics statement

The analysis was granted exemption status from the University of Washington Institutional Review Board (IRB) under a minimal risk determination status. The analysis was determined not to be human subjects’ research based upon the self-determination process at the University of Washington IRB. Verbal consent was obtained from all individual participants in surveys and focus group discussions (FGDs). All survey data was de-identified to ensure anonymity and qualitative transcripts were reviewed prior to analysis to ensure that they do not contain any identifiable information linked to individual participants.

### Study design

This study used a convergent mixed-methods design, collecting quantitative data from online surveys and qualitative data from FGD in parallel. Integration occurred during data analysis and interpretation of results.

### Sampling and recruitment

Study participants included MOH NTD PMs from NTD-endemic countries across sub-Saharan Africa and their implementing partners from non-governmental organizations (NGOs) working on NTDs. Both stakeholder groups were recruited using purposive sampling techniques. PMs were recruited from a membership database of regional PMs and NGO representatives recruited from the Neglected Tropical Disease NGO Network (NNN) listserv. In total, 48 PMs and 1,974 NGO representatives were invited to complete the survey via email. All 48 PMs were also invited to participate in FGDs via email invitations.

### Data collection

The online survey was developed and administered by WHO Regional Office for Africa through the Expanded Special Project for Elimination of Neglected Tropical Diseases (ESPEN). The survey included four main questions and 11 sub-questions, with a mixture of closed and open-ended responses (S1 Appendix). Questions were related to the status of the MDA program for each PC-NTD (started, plan to restart, or no plan to restart); challenges associated with restarting MDA for key implementation activities (e.g., drug supply chain, training, community engagement, drug administration, and monitoring & evaluation) and required resources (e.g., workload and staffing, funding, incorporation of COVID-19 safety guidelines). The survey also asked respondents to identify any proposed solutions or innovations for restarting MDA programs. innovations to MDA delivery that might accelerate progress towards disease control and elimination benchmarks upon reopening. Administration of the survey took place between August 8 and September 18, 2020.

Three FGDs were conducted with PMs, one each in English, French, and Portuguese. The FGDs took place over 60-minute Zoom calls (S2 Appendix) and were recorded with the permission of participants. All recordings were transcribed and French and Portuguese transcripts were translated into English by private translation companies. Transcripts were uploaded to ATLAS.ti version 22.09 [8]. After transcripts were read in full, a combined inductive and deductive approach was used to develop a codebook. The codebook was used by a single coder to code each of the three transcripts, with a second coder consulted when necessary. Once the coding process was complete, memos were written to summarize main findings followed by a thematic analysis to identify a key themes.

### Data analysis

Descriptive analyses were used to present response counts and proportions for close-ended questions, separately for PMs and NGO respondents. Open-ended questions were organized into common responses categories. Survey data were analyzed in Stata version 13.1 [9]. Triangulation was used for data integration to determine convergence or divergence across the quantitative and qualitative findings [10]. This process generated a deeper understanding of the strength of specific findings.

## Results

In total, 10 PMs from 10 countries and 37 NGO representatives from 25 organizations responded to the online survey (21% and 2% response rate, respectively). Survey respondents participated in MDA programs of all five PC-NTDs (Table 1). Despite MDA program activities being halted due to the WHO guidance, over 40% of PMs and NGO participants reported plans for MDA activities to restart for all five PC-NTDs in 6–12 months after the survey was administered (Table 1). A total of 37 NTD PMs from 17 countries participated in the FGDs (77% response rate).

**Table 1:** PM and NGO participant survey results. Shows all survey responses to each question from 10 PMs from 10 countries and 37 NGO representatives.

Description		PMs (N = 10) n (%)	NGOs (N = 37) n (%)
**PC-NTD programs represented by survey respondents** ^**1**^			
Lymphatic filariasis		8 (80)	28 (76)
Onchocerciasis		7 (70)	24 (65)
Schistosomiasis		10 (100)	24 (65)
Soil-transmitted helminths		9 (90)	23 (62)
Trachoma		8 (80)	23 (62)
**Plans to re-start or have re-started MDAs** ^**1**^			
Lymphatic filariasis	Started	2 (25)	6 (24)
	Plan to re-start	6 (75)	20 (71)
	No plan to re-start	0 (0)	2 (7)
Onchocerciasis	Started	0 (0)	6 (25)
	Plan to re-start	7 (100)	17 (71)
	No plan to re-start	0 (0)	1 (4)
Schistosomiasis	Started	3 (30)	8 (33)
	Plan to re-start	7 (70)	16 (66)
	No plan to re-start	0 (0)	0 (0)
Soil-transmitted helminths	Started	2 (22)	6 (26)
	Plan to re-start	7 (78)	17 (74)
	No plan to re-start	0 (0)	0 (0)
Trachoma	Started	2 (25)	7 (30)
	Plan to re-start	4 (50)	16 (42)
	No plan to re-start	1 (12)	0 (0)
**Experienced interruption of prevalence surveys**		7 (70)	35 (94)
**Plan to use mobile technology to collect data**		4 (40)	10 (27)

^1^ The denominator for each proportion is the number of respondents in each endemic area. Responses are not exclusive; some respondents mentioned more than one NTD.

Survey and FGD results coalesced around six themes. The first three themes identified pandemic-related factors that caused disruptions to MDA program delivery, including 1) diversion of financial and to the pandemic response, 2) challenges adhering to COVID-19 safety protocols, and 3) community hesitancy due to coronavirus transmission fears. The last three themes detailed potential solutions and proposed innovations for relaunching MDA programs amidst the pandemic, namely 4) adapting intervention packaging and delivery mechanisms to minimize COVID transmission, 5) embracing technology to optimize intervention planning and delivery, and 6) identifying opportunities to promote program integration between pandemic response strategies and NTD campaign delivery.

### Disruptions to MDA program delivery

**Fig 1:**
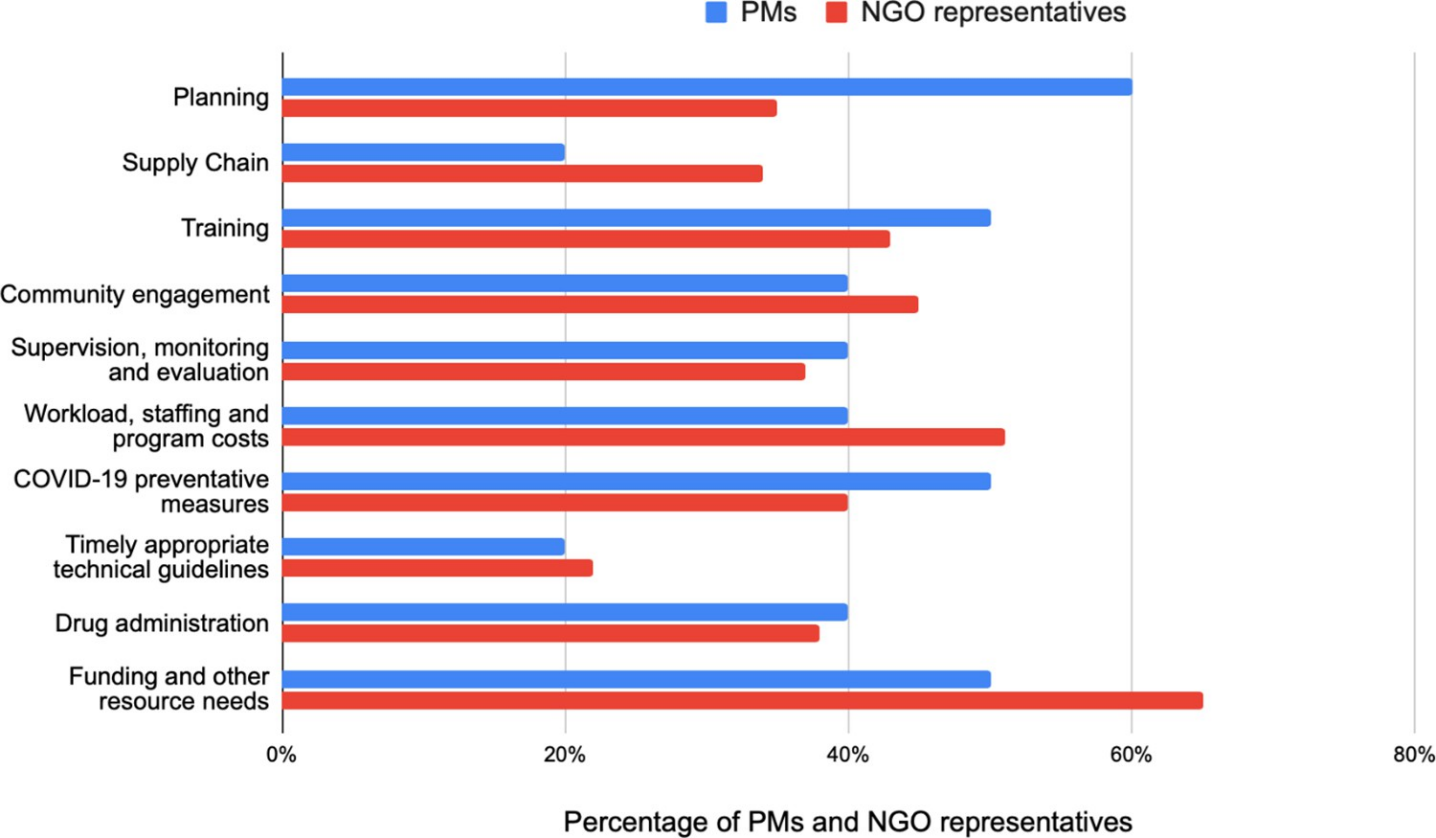
Disruptions to specific MDA program activities due to COVID-19. Shows responses from NGO and PMs participants specifically on MDA program activities that had been disrupted due to COVID-19.

Fig 1 displays the key disruptions to specific MDA program activities due to COVID-19, as indicated by survey respondents.

#### Diversion of financial and human resources to the pandemic response created shortages for NTD programs

The most commonly reported challenges resulting from WHO guidance on postponing MDA activities were related to resource needs. Specifically, PMs and NGO respondents indicated having financial and human resource gaps (50% and 65%, respectively), as these resources were being diverted from NTD programs to the pandemic response. PMs further described how efforts to restart MDA activities during the pandemic would require additional resources to adhere to COVID-19 safety measures:

“Financial support might be required to provide masks, hand-washing facilities and alcohol based hand sanitizer.”**—PM #3**

“Even if we have to launch MDAs now, there needs to be a basic level of safety and the logistics of this generate extra costs compared to what was previously planned.”—**PM #5**

These resource gaps further affected program workload, staffing and program costs, as indicted by 50% and 45% of PMs and NGO respondents, respectively. During FGDs, PMs indicated how there would be need to reduce the number of CDDs, which would have further impacts on the number of days of drug distribution.

“We shall try and change the strategy or model the campaign to avoid crowds of people in the classrooms. We shall try and cut the number of community health agents and no doubt the days of the campaign will be altered.”—**PM#11**

#### Challenges adhering to COVID-19 safety protocols disrupted training, community engagement, and monitoring activities

Nearly half of all PMs and NGO respondents (50% and 40%, respectively) reported challenges adhering to COVID-19 safety measures such as maintaining physical distancing or procuring protective equipment (PPE) (Fig 1). Specifically, PMs and NGO respondents indicated that COVID safety protocols directly affected CDD trainings (50% and 45%, respectively), and community engagement and sensitization activities (40% and 45%, respectively). They noted that trainings were normally facilitated in-person but had to be delayed due to physical distancing measures. These sentiments were echoed in the FGDs, as PMs described challenges for CDDs and other health workers in maintaining physical distancing among large community gatherings such as public community sensitization activities.

“In [COUNTRY REDACTED], our community engagement strategy requires that our administrators congregates a number of constituents within the areas where the they are in charge and then pass the information to them … now … that particular methodology may not work [because of physical distancing].”—**PM #1**

In addition to the disruptions to training and community mobilization, PMs and NGO respondents also indicated interferences on monitoring and evaluation activities (70% and 94%, respectively), specifically on planned prevalence and coverage surveys.

“When we were getting ready to organize our coverage survey and our meeting to evaluate activities in 2019…we had to cancel the surveys and the meetings”—**PM #3**

They noted how these delays disrupted the program’s ability to determine the populations at risk, which had further implications on the drug supply chain.

“Because of COVID they can’t do granular mapping for more precise treatment and drugs are about to expire”—**PM #12**

#### Coronavirus transmission fears drove community and health worker hesitancy to participate in MDA campaigns

**Fig 2:**
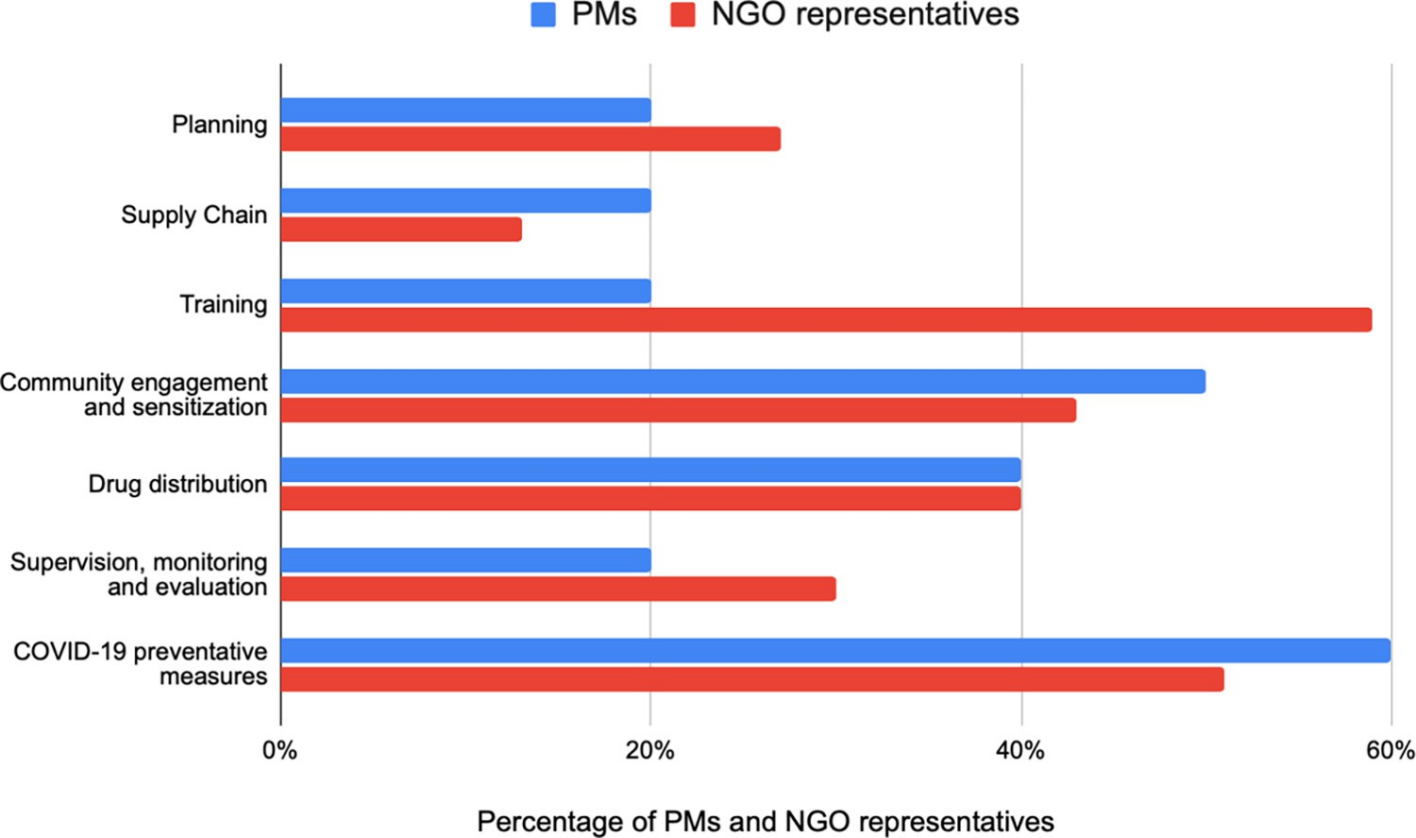
MDA program activities that require adjustment to relaunch MDA during COVID-19. Shows responses from NGO and PMs participants specifically on program activities that need support and require adjustment when relaunching MDA during COVID-19.

A primary challenge for restarting MDA delivery centered around concerns about coronavirus transmission during MDA activities, as 40% of both PMs and NGO respondents regarded that drug distribution would require significant adjustments prior to program relaunch (Fig 2). PMs elaborated upon this concern in FGDs, where they described community concerns about coronavirus transmission during MDA activities.

“But the most important thing isn’t these resources—it’s convincing the community to join the campaign because the COVID phenomenon has brought with it a kind of psychosis, a fear of the unknown. People are afraid of everything different to what they already know. They’re scared of catching the virus from people who come to supervise or community liaison officers who come to the village to help them.”—**PM #12**

“In the midst of COVID, many communities are afraid that health workers are the ones carrying disease into their communities.”—**PM #7**

### Solutions for restarting MDA programs

Fig 2 displays the specific MDA delivery activities that respondents indicated would require adjustments prior to program restart.

#### Adapting intervention packaging, sensitization, and delivery strategies to complement pandemic safety precautions

Respondents proposed certain adaptations around intervention design and packaging to abide by coronavirus safety precautions. One adaptation recommended prioritizing the use of spoons or providing the drugs in premeasured envelopes to minimize contact during drug administration. One PM in the FGDs also suggested adaptations specifically for using the dosage pole to administer schistosomiasis treatment.

“You can put the dose pole on the wall, and have the person go stand next to it. The CDD can look at the measurement, while still maintaining distancing.”—**PM #1**

In light of community member fears about COVID-19 transmission, PMs and NGO participants suggested adapting community sensitization messages that respond to the specific coronavirus transmission concerns (50% and 43%, respectively). During the FGDs, PMs proposed that community sensitization methods could make use of existing practices such as using public address systems mounted on vehicles to allow for physical distancing and to embed health education messages about coronavirus during community engagement activities. However, they noted that there may be resource challenges associated with these adaptations, given the need to scale up the approaches to reach more community members.

“As it may limit transmission of COVID-19, we are looking into alternative strategies of sensitizing and mobilizing the community through use of public address system mounted on a moving vehicle. So this comes with a challenge of getting [enough] vehicles and personnel to adequately conduct the movement … and reach the population.”—**PM #1**

PMs and NGO representatives also suggested that programs adjust drug distribution strategies to mitigate risk of COVID-19 transmission (40% for both groups) (Fig 2). PMs suggested prioritizing door-to-door drug delivery (as opposed to fixed-point delivery) as a way to control and maintain physical distancing. However, during FGDs, PMs noted that to conduct door-to-door delivery, CDDs might require additional incentives, given potential personal hazards of conducting MDA during the pandemic.

“Another challenge I could anticipate, seeing as our community health workers go door to door to dispense the drugs, would be whether they are going to need a greater incentive given the risks they are taking”—**PM #6**

#### Embracing technology to optimize program planning and data collection

Survey participants suggested the importance of embracing technology innovations, some introduced during the pandemic, throughout intervention planning and delivery activities. For example, PMs (40%) and NGO participants (27%) reported plans to use mobile technology to collect data individual- and household-level coverage data, rather than using time-consuming paper-based methods that are often challenging to aggregate and prone to data errors. In addition to improve upon NTD monitoring & evaluation activities, PMs suggested in the FGDs how mobile data collection and other online technology could be used to not minimize the risk of COVID-19 transmission for key stakeholders involved in MDA delivery, but also streamline key intervention activities and potentially reduce program delivery costs. For example, PMs reported plans to use online meeting platforms for training health workers and conducting planning meetings.

“We must begin to think about what we have to do to make our work easier. We have thought about online training. We can also think about digital data collection via mobile phone…I think that we can also have multiple online meetings and there is nothing stopping us teaching the people involved in implementing activities how to use these new tools. I think that the easier it is, the more things we could organize smoothly while minimizing the risk involved in carrying out our activities.”—**PM #3**

#### Promoting NTD program integration with pandemic response activities

Survey participants commonly proposed integrating NTD program activities with other health programs to help pool financial and human resources together during the pandemic and decrease the risk of COVID-19 transmission, thus optimizing program delivery.

“Integrating is one approach to minimize contact, time in the community, as well as to integrating activities while resources are stretched thin”—**PM #7**

PMs also discussed the feasibility of integration NTD delivery with ongoing community programs such as malaria or immunization campaigns and highlighted the importance of having support from local Ministry of Health.

“There is a malaria spray project where we are assessing the possibility of integrating [NTD delivery] with this project, given that the spraying will also be done door-to-door in this area, in these border municipalities. This province has many integration initiatives already, through these projects, [including] the extended vaccination programme … The provincial Department of Health is favouring integration.” **PM #8**

Similarly, PMs suggested leveraging NTD programs for co-delivery of COVID-19 prevention and control. They noted that MDA platforms have established transportation and human resource chains to rural areas that can be used for COVID-19 education, case detection, and contact tracing. Additionally, PMs discussed how COVID-19 initiatives could be used as platforms for sensitizing community members about NTDs, which could help build community trust towards these programs.

“In these COVID-related initiatives, we included NTD messages to show people that NTD interventions are still here to support them during the pandemic.” **PM #12**

## Discussion

In this study, PMs and NGO partners were frequently optimistic about the prospects of restarting MDA program activities, despite the implementation delays caused by the pandemic. Participants highlighted how safety protocol-related challenges disrupted key MDA activities, including prevalence assessments, community sensitization activities, implementer trainings, drug distribution, and coverage surveys. They also identified several solutions to mitigate these disruptions, including adapting intervention delivery to lessen ongoing coronavirus transmission, as suggested in the WHO Risk Assessment Tool [11]. Some recommendations recognized existing MDA practices for drug delivery such as utilizing door-to-door treatment rather than fixed-point delivery and using spoons or pre-packaged drugs to avoid direct handling of medicines, all which align with strategies in recently published studies highlighting best practices for delivering MDA during the pandemic [12–15].

Participants also discussed embracing technology to optimize intervention planning and delivery. Although the use of mobile technology is heavily covered in the current NTD literature as important strategies for effectively MDA delivery [16–20], this study further continues the discussion around the feasibility of mobile technology for epidemiologic data collection during public health emergencies [21–23], as discussed in recent studies conducted in sub-Saharan Africa during ongoing pandemics, including COVID-19 and Ebola [21,23–25]. In addition to promoting health worker and community safety, mobile data collection during a pandemic offers important advantages, including flexibility and responsivity to evolving data needs, improved timeliness and cost-effectiveness of data collection, and improved utility for monitoring and evaluation procedures [24]. These technology discussions further align with participants noting the opportunity to integrate NTD efforts into pandemic response programs. Several studies have highlighted the effectiveness of program integration, specifically regarding higher coverage, increased cost-effectiveness, and optimized operational convenience [26–31]. Recent research further demonstrates the potential for NTD programs to serve as a platform for pandemic preparedness, illuminating the gains in program efficiency, platform resiliency, and building multisectoral collaborative efforts [11,15,32–34]. Therefore, as NTD programs relaunch, there is an opportunity to integrate program activities that limit field activities in communities, maximize use of limited resources, and optimize coverage–efforts that align with recommendations in the WHO 2030 NTD Road Map to accelerate efforts to implement sustainable NTD programming [7]. For example, CDDs who participate in MDAs can support COVID-19 vaccine uptake to further incentivize integrated programming across NTDs and other health programs, including vitamin A distribution, malaria treatment, polio immunization, and nutrition campaigns [35–39].

Participants in this study also emphasized the importance of creating tailored community sensitization plans that respond to the specific priorities and concerns of community members regarding COVID-19 transmission. Respondents noted that community members were often fearful of coming in contact with CDDs; thus, engagement strategies not only needed to be adapted to ensure physical distancing, but also to allay community hesitancy. The suggestion provided by PMs to utilize public service announcements to minimize close contact and to embed messages regarding coronavirus transmission into NTD health education strategies align with other literature highlighting best practices for community engagement when restarting MDA during pandemics [12–15]. Tailoring community sensitization messages will require engaging with communities to understand their concerns and adapt sensitization messages and delivery platforms accordingly, as evidence during the 2015 Ebola outbreak in Liberia, where researchers surveyed community leaders to develop tailored health messaging to address community concerns e.g. highlighting that there is no linkage between MDA and Ebola transmission [40]. Thus, integrated promotional messaging combined with practical changes to MDA activities that promote safety precautions could help to overcome fears surrounding MDA during a pandemic.

### Strengths and limitations

Findings from our study provide useful information related to the resumption of community health programs that have been disrupted by COVID-19. Notably, the status of many MDA programs in NTD-endemic countries in sub-Saharan Africa may have changed since the time this data was collected; however, these findings continue to remain relevant, given the ongoing nature of the pandemic, which continues to impact public health delivery globally. Furthermore, given the small sample size of the survey results, the findings may not be generalizable and accurately represent opinions of all PMs from African NTD-endemic countries or all NGO representatives working on NTD program activities in the sub-Saharan region. However, our respondents did include a diverse representation of countries and organizations. With these limitations in mind, this paper provides additional contribution to existing commentaries that could guide countries on the resumption of MDA and other NTD program activities disrupted by global pandemics. [34,41,42].

## Conclusion

This study identified challenges to implementing MDA during the COVID-19 pandemic, and solicited recommendations and innovations for how to safely restart MDA. The proposed solutions may help address major disruptions such as implementer and community fears to engage in MDA activities as well as resource shortages that reduce the ability to incorporate pandemic preventative measures into NTD programs. Thus, the significant investments needed to relaunch paused programs should also be used to catalyze innovations to MDA delivery, helping programs both to overcome the significant hurdles presented by the COVID-19 pandemic as well as accelerate NTD program achievements moving forward.

## Supporting information

S1 Appendix Online questionnaire for National Program Managers and Non-Governmental Organization Stakeholders to understand the challenges and opportunities of MDA programs in the context of COVID-19. (DOCX)

S2 Appendix Focus Group Discussion Interview Guide. (DOCX)

S1 Data Quantative data NGO participants. (CSV)

S2 Data NTD Program Managers quantitative data. (CSV)
