# Supplementary material for: “Our interventions are still here to support communities during the pandemic”: Resuming mass drug administration for neglected tropical diseases after COVID-19 implementation delays
Source: PLoS Negl Trop Dis. 2023 Jun 26;17(6):e0011368. doi: 10.1371/journal.pntd.0011368 (PMC10328222; doi:10.1371/journal.pntd.0011368)
Supplement: S1 Appendix — (DOCX) [file pntd.0011368.s001.docx]

**Online questionnaire for National Program Managers and Non-Governmental Organization Stakeholders to understand the challenges and opportunities of MDA programs in the context of COVID-19**

| **A: Brief description of country Preventative Chemotherapy (PC) -NTD program and impact of COVID-19 on MDAs** | | | | | |
| --- | --- | --- | --- | --- | --- |
| Question 1: For each PC-NTD, please answer the following; | | | | | |
|  | Lymphatic filariasis | Onchocerciasis | Schisto-somiasis | Soil Transmitted Helminths (STH) | Trachoma |
| 1.1: Endemic diseases (Yes/No) |  |  |  |  |  |
| 1.2: Delivery platform (**1-school-based, 2-community-based, 3-fixed post, 4-mixed**) |  |  |  |  |  |
| Follow disease columns from top and enter **1-3** |  |  |  |  |  |
| 1.3: Based on the experience of missed or delayed rounds in the past, how concerned are you about the impact of current MDA delays on the progress towards control and elimination goals? **1 - No concern, 2 – Some concern, 3 – Large concern** | | | | | |
| Follow disease columns from top and enter **1-3** |  |  |  |  |  |
| 1.4: Do you have plans to re-start or have re-started MDAs for the different diseases?  **1-started, 2-plan to re-start, 3-no plan to re-start** | | | | | |
| Follow disease columns from top and enter **1-3** |  |  |  |  |  |

| **B: Understanding challenges associated with re-starting MDA and other interventions, and proposed solutions** | | |
| --- | --- | --- |
| Question 2: How has COVID-19 disrupted NTD program activities in your country?  **1-No impact, 2-Minimal impact, 3-Large impact**  Where you score 3, please explain how COVID-19 has disrupted activity | | |
| **Activity** | **Score (1-3)** | **Explanation** |
| 2.1: Planning (national and local planning) |  |  |
| 2.2: Supply chain (drug transportation and availability at local levels for distribution) |  |  |
| 2.3: Training (content, timing, availability of trainers and participants) |  |  |
| 2.4: Community engagement and sensitization (CDD engagement and safety, messaging, and use of mass and social media etc) |  |  |
| 2.5: Drug administration (delivery strategy, dose determination, dosing and recording) |  |  |
| 2.6: Supervision, monitoring and evaluation |  |  |
| 2.7: Workload, staffing requirements and program costs |  |  |
| 2.8: Practical ability to incorporate and adhere to COVID-10 measures (PPEs, social distancing, handwashing, reassignment of program staff to COVID-19) |  |  |
| 2.9: Timely and appropriate technical guidelines and integrated SOPs (involving COVID-19 and MDAs, practical application) |  |  |
| 2.10: Funding and other resource needs |  |  |
| 2.11: Has COVID-19 made you change activities which will be beneficial for achieving program goals? Yes/No |  |  |
| 2.14: For the main challenges above, what solutions/changes do you propose for addressing the most impactful challenges associated with restarting MDA? | | |
| **Challenge (2.x)** | **Proposed solution(s)** | |
| 1. |  | |
| 2. |  | |
| 3. |  | |
| 4. |  | |

| **C: Impact of COVID-19 on plans for epidemiological surveys and potential challenges.** | |
| --- | --- |
| - 1. Have plans for epidemiological surveys been disrupted by COVID-19? (Y/N) |  |
| - 1. In what ways have the plans been updated? |  |
| - 1. What changes will you make to how you undertake impact surveys, especially to minimize risk of SARS-Cov2 transmission? |  |
| - 1. What specific changes will you make to collect and process samples safely? |  |
| - 1. Will you combine different disease-specific surveys? If so, how? |  |
| - 1. Will you use mobile technology (e.g. smart phones or tablets) to co**l**lect data? If so, for which diseases? Has this changed from before? |  |

| **D: Potential impact of innovation** | | |
| --- | --- | --- |
| Question 4: For which program activities will new and innovative ideas be most impactful in the delivery of MDA and other NTD intervention in a context of COVID-19?  Indicate the importance of introducing change on a scale of 1-3  **1-Not important, 2-Important, 3-Very important**  Where you score 3, please explain reason | | |
| **Activity** | **Score (1-3)** | **Explanation** |
| 4.1: Planning (national and local) |  |  |
| 4.2: Supply chain (drug availability from national to community level for distribution) |  |  |
| 4.3: Training |  |  |
| 4.4: Community engagement and sensitization |  |  |
| 4.5: Drug distribution |  |  |
| 4.6 Supervision, monitoring and evaluation |  |  |
| 4.7: COVID-10 measures (PPEs, social distancing, handwashing etc.) |  |  |

| F: Opportunities for improvement | |
| --- | --- |
| Question 5: Aside from increased funding for delivery, what improvements would you like to see for improving the planning, implementation and evaluation of NTD programs in the context of COVID-19? | |
| Opportunity 1 |  |
| Opportunity 2 |  |
| Opportunity 3 |  |
| Opportunity 4 |  |
