# Supplementary material for: “Our interventions are still here to support communities during the pandemic”: Resuming mass drug administration for neglected tropical diseases after COVID-19 implementation delays
Source: PLoS Negl Trop Dis. 2023 Jun 26;17(6):e0011368. doi: 10.1371/journal.pntd.0011368 (PMC10328222; doi:10.1371/journal.pntd.0011368)
Supplement: S2 Appendix — (DOCX) [file pntd.0011368.s002.docx]

Restarting Mass Drug Administration for Neglected Tropical Disease Programs during the COVID-19 pandemic

**Focus Group Discussion Interview Guide:**

COVID-19 has changed the context in which NTD programs are implemented and brought new challenges. Today we are interested to hear about your experience and the challenges you have faced. As you look to resume NTD activities, there are important ways NTD programs may need to evolve in a new context with COVID-19. For example, the way MDAs and surveys are conducted will need to be adapted to accommodate the shift to door-to-door activities. There may be opportunities to combine delivery of NTD interventions with other NTD interventions and with other health programs.

The progress of some programs may have been adversely affected by the pausing of interventions, and alternative intervention strategies may be required to kick-start and accelerate progress towards 2030 goals. Lastly, innovative approaches and tools will be required to address some of these challenges – some of these innovations may be digital, some may be imaginative innovative ways of conducting activities.

To help identify innovative solutions, the Bill & Melinda Gates Foundation in partnership with other NTD funders are planning a series of meetings during the month of September when the NTD community comes together to present, discuss and develop such innovations. Further details of the series and funding will be made available later this month but, in brief, we envisage to award in 2020 a series of medium-sized grants of $200-300,000 and for exceptional ideas to provide larger amounts of funding.

The funding is intended to support new approaches and tools. The funding is not to cover the acknowledged increased costs due to PPE or extra staff; these costs should be covered from your implementation funding.

We are grateful for the insights some of you have already provided through the ESPEN online survey, today we wish to build on the survey and delve deeper into the challenges your programs face and the kind of solutions you would wish to see address them.
